# Progranulin induces immune escape in breast cancer via up-regulating PD-L1 expression on tumor-associated macrophages (TAMs) and promoting CD8^+^ T cell exclusion

**DOI:** 10.1186/s13046-020-01786-6

**Published:** 2021-01-04

**Authors:** Wenli Fang, Ting Zhou, He Shi, Mengli Yao, Dian Zhang, Husun Qian, Qian Zeng, Yange Wang, Fangfang Jin, Chengsen Chai, Tingmei Chen

**Affiliations:** Key Laboratory of Clinical Laboratory Diagnostics (Ministry of Education), College of Laboratory Medicine, Chongqing Medical University, Chongqing, 400016 People’s Republic of China

**Keywords:** Breast cancer, Progranulin (PGRN), TAMs, PD-L1, T cell

## Abstract

**Background:**

Progranulin (PGRN), as a multifunctional growth factor, is overexpressed in multiple tumors, but the role of PGRN on tumor immunity is still unclear. Here, we studied the effect of PGRN on breast cancer tumor immunity and its possible molecular mechanism.

**Methods:**

The changes of macrophage phenotypes after PGRN treatment were detected by western blot, quantitative polymerase chain reaction (PCR) and flow cytometry. Western blot was used to study the signal molecular mechanism of PGRN regulating this process. The number and localization of immune cells in Wild-type (WT) and PGRN^−/−^ breast cancer tissues were analyzed by immunohistochemical staining and immunofluorescence techniques. The activation and proliferation of CD8^+^ T cells were measured by flow cytometry.

**Results:**

After being treated with PGRN, the expressions of M2 markers and programmed death ligand 1 (PD-L1) on macrophages increased significantly. Signal transducer and activator of transcription 3 (STAT3) signaling pathway inhibitor Stattic significantly inhibited the expression of PD-L1 and M2 related markers induced by PGRN. In WT group, CD8 were co-localized with macrophages and PD-L1, but not tumor cells. The number of immune cells in PGRN^−/−^ breast cancer tissue increased, and their infiltration into tumor parenchyma was also enhanced. Moreover, in the co-culture system, WT peritoneal macrophages not only reduced the ratio of activated CD8^+^ T cells but also reduced the proportion of proliferating CD8^+^ T cells. The addition of programmed death receptor 1 (PD-1) and PD-L1 neutralizing antibodies effectively reversed this effect and restored the immune function of CD8^+^ T cells.

**Conclusion:**

These results demonstrate that PGRN promotes M2 polarization and PD-L1 expression by activating the STAT3 signaling pathway. Furthermore, through PD-1/PD-L1 interaction, PGRN can promote the breast tumor immune escape. Our research may provide new ideas and targets for clinical breast cancer immunotherapy.

**Supplementary Information:**

The online version contains supplementary material available at 10.1186/s13046-020-01786-6.

## Introduction

In recent years, as an emerging treatment, breast cancer immunotherapy has attracted tons of attention [1], especially systemic therapies mediated by programmed death ligand 1 (PD-L1) [2]. However, the immune escape generated during treatment raises a new challenge to its clinical efficacy [3]. Therefore, it is very urgent to study the potential effect factors that regulate PD-L1. PD-L1 can cause T cell dysfunction and failure, prevents cytotoxic T cells from effectively targeting tumor cells via binding to the programmed death receptor 1 (PD-1) on T cells, and thus promotes the occurrence and development of tumors [4, 5]. Recently, research on PD-L1 has mainly focused on tumor cells [6]. For instance, an immunosuppressive microenvironment characterized by elevated PD-L1 was observed in a lung tumor model driven by the epidermal growth factor receptor (EGFR) pathway [7]. However, interestingly, there have been reported that tumor-associated macrophages (TAMs) are the main source of PD-L1 expression in the tumor microenvironment (TME). The expression of PD-L1 on TAMs is more sustaining than those on tumor cells [8]. Unfortunately, the research on the regulatory factors of PD-L1 on TAMs has not been fully elucidated.

Tumor-associated macrophages, as an important component of tumor infiltrating immune cells, play a crucial part in tumor invasion and metastasis [9, 10]. According to different activation signals, they are mainly classified as M1 and M2 phenotype. Usually, TAMs are dominated by M2 macrophages that promotes tumor progression in the tumor microenvironment [11, 12]. M2 macrophages participate in tumor growth, invasion and metastasis and angiogenesis by secreting cytokines and growth factors, meanwhile immunosuppressive cytokines released by M2 macrophages inhibit the function and metabolism of T cells [13, 14]. In tumor immunity, CD8^+^ T cells are the key tumor-suppressing cells by forming physical contact with malignant tumor cells and inducing the death of tumor cells through activating their intracellular signals [15, 16]. Current studies have found that the occurrence of “immune exclusion” in the tumor microenvironment is a key factor inhibiting cancer immunotherapy. Detailly, CD8^+^ T cells are excluded from the vicinity of cancer cells and their immune function is suppressed, which results in the lack of CD8^+^ T cells in the tumor microenvironment and immune evasion of tumor cells [17–19]. However, the molecular mechanism that causes T cell exclusion in breast cancer has not yet been fully defined. Of interest, Recent studies have reported that progranulin (PGRN) also has something to do with the macrophage polarization [20, 21]. However, there is little report about how PGRN affects the polarization and function of macrophages in breast cancer. And whether PGRN can affect tumor immunity through macrophages is also urgently needed to be studied.

Progranulin (PGRN) is composed of 593 amino acids [22], and participates in various pathophysiological processes including neurodegeneration, tissue damage repair, embryonic development and so on [23]. In the past, people paid more attention to the role of PGRN in inflammatory diseases [24]. But recently, researchers have shifted their attention to the relationship between PGRN and tumors [25]. Studies have reported that PGRN is relevant to the poor prognosis of patients with tumor [26]. Unfortunately, so far, the effect of PGRN on the development of breast cancer and the specific molecular mechanism have not been fully determined.

This study is the first time to clarify the effect of PGRN on PD-L1 expression in TAMs rather than tumor cells. We found a novel mechanism for PGRN to promote breast cancer immune escape. By focusing on macrophages, we demonstrated that PGRN up-regulated the PD-L1 expression of TAMs through JAK/STAT3 signaling pathway and promoted the polarization of TAMs to M2 macrophages. Then PGRN-treated macrophages mainly inhibited T cell proliferation and its killing activity through the interaction of PD-1/PD-L1, and resulted in CD8^+^ T cell exclusion. Overall, these findings further clarified the immunosuppressive function of TAMs. In addition, we aimed to reveal the crucial function of PGRN on inducing immunosuppression in breast cancer, hoping that this study could provide new ideas for enhancing the clinical immune efficacy of breast cancer.

## Methods

### Cell lines and reagents

Raw264.7 cells were cultured in complete DMEM (Gibco) medium. PY8119 cells were cultured in DMEM:F12(1,1) (Gibco) supplemented with 10% FBS (Gibco). As previously described [27, 28], macrophages were polarized in M1 macrophages with 100 ng/mL LPS (Sigma, USA, L2630) and 20 ng/mL IFN-γ (R&D Systems, USA, 485-MI-100); and macrophages were treated with 20 ng/mL IL-4 (NOVUS, USA, NBP2–35131) to generate M2 macrophages.

Small interfering RNA (siRNA) (Invitrogen) for PGRN was transfected with R4000 (Engreen, China, 4000–4). Recombinant murine PGRN was purchased from RD (R&D Systems, USA, 2557-PG). Stattic (HY-13818), LY294002 (HY-10108) and U0126 (HY-12031A) were purchased from MCE (Shanghai, China).

### Peritoneal macrophages isolation

Peritoneal macrophages were generated according to previous literature [29]. Briefly, each C57BL/6 mouse was injected intraperitoneally with 2 ml of 3% thioglycollate (Difco) on day 1. After administration, the mice were kept under daily observation to check for abnormal activities. Mice were sacrificed 4 days after injection. Peritoneal macrophages were harvested from peritoneum, after being injected with 7 ml cold PBS into peritoneal cavity. The peritoneal cells were centrifuged at 1500 RPM for 10 min then seeded into cell culture dishes. The suspension cells were discarded by washing with PBS after 2 h. And the adherent cells were considered as peritoneal macrophages.

### In vitro T cell activation assay and co-culture

According to previous literature [30], briefly, spleens were ground with syringes, washed with PBS, and then passed through 70 μm cell strainers to gain single-cell suspensions. Red blood cells were lysed by using Red Blood Cell Lysing Buffer (Biosharp, China, BL503B). Splenocytes were further separated with C57BL/6 mouse spleen lymphocyte separation solution (tbdscience, China, LTS1092PK) to obtain spleen lymphocytes. Obtained spleen lymphocytes were cultured in complete RPMI 1640 medium. For T-cell activation assays, anti-CD3e (5 μg/ml; eBioscience, 16–0031-82) was pre-coated in 96 well plates overnight at 4 °C. Subsequently anti-CD28 (1 μg/ml; eBioscience, 16–0281-82) was added to the plates.

For co-culture assay, peritoneal macrophages at indicated ratios were added to the medium after T cell activation. Moreover, cells were cocultured with or without neutralizing monoclonal antibodies against PD-L1 (eBioscience, 16–5982-82), PD-1 (BioXcell, BP0273) or IgG isotype control. After 4 days, cells were detected by flow cytometry.

### Immunohistochemistry

Sections from tumors were cut into 4 μm in thickness and deparaffinized in xylene for 10 min. The slides were immersed in 3% H_2_O_2_ for 20 min to block the endogenous peroxidase and were blocked in goat serum blocking solution for 30 min. After being incubated at 4 °C overnight with primary antibodies, the slides were incubated with secondary HRP-conjugated antibodies (Thermo Fisher Scientific) for 30 min at RT. The primary antibodies were as follows: F4/80 (Cell Signaling Technology, 70076S, 1:200), iNOS (Abcam, ab3523, 1:50), CD206 (proteintech, 18,704–1-AP, 1:200), CD4 (Cell Signaling Technology, 25229S, 1:200), CD8 (Cell Signaling Technology, 98941S, 1:200), Granzyme B (NOVUS, NB100–684, 1:200), and PD-1 (Abcam, ab214421, 1:200). IHC stainings were examined with microscopy.

### Immunofluorescence

Slides from tumors were deparaffinized in xylene and dehydrated in graded ethanol solutions. The sections were blocked in goat serum blocking solution for 1 h at room temperature. The slides were incubated overnight at 4 °C with the following antibodies for multicolor immunofluorescence staining: F4/80 (Cell Signaling Technology, 30,325 T, 1:200), PD-L1 (Abcam, ab213480, 1:100), CD206 (proteintech, 18,704–1-AP, 1:200), Arg1 (Abcam, ab96183, 1:100), iNOS (Abcam, ab178945, 1:200), CD8 (Bioss, bs0648R, 1:100), CD4 (Bioss, bs-0647R, 1:100), CK19 (Abcam, ab52625, 1:200), and PD-1 (Abcam, ab214421, 1:100). The next day, the slides were washed in PBS and stained with the secondary antibody for 1 h at room temperature. Multicolor immunofluorescence staining was detected with fluorescence microscope.

### Western blot

The cells were lysed in RIPA lysis buffer (Beyotime, China, P0013B) supplemented with protease inhibitors PMSF (Beyotime, China, ST506). Protein concentration were determined by using BCA protein assay kit (Beyotime, China, P0012S). The primary antibodies included iNOS (Abcam, ab178945, 1:1000), Arg1 (Abcam, ab233548, 1:2000), PGRN (Abcam, ab187070, 1:1000), PD-L1 (Abcam, ab213480, 1:1000), STAT3 (Cell Signaling Technology, 9139 T, 1:1000), pSTAT3 (Cell Signaling Technology, 9145 T, 1:1000), AKT (Cell Signaling Technology, 4685S, 1:1000), pAKT (Cell Signaling Technology, 4060 T, 1:1000), ERK1/2 (Cell Signaling Technology, 9102S, 1:1000), pERK1/2 (Cell Signaling Technology, 4377 T, 1:1000), and β-actin (proteintech, 66,009–1-Ig, 1:2000).

### RNA extraction and real-time quantitative PCR

Total RNA was extracted with TRIzol reagent (TaKaRa, Japan, 9109), and cDNA was reverse transcribed subsequently with PrimeScript RT reagent kit (TaKaRa, Japan, RR037A). qRT-PCR was then performed using a SYBR Premix Ex Taq II (TaKaRa, Japan, RR820A) according to the manufacturer’s instructions. The sequences of primers were presented in Table 1.

**Table 1:** Primers used for qRT-PCR

Gene name	Forward (5′-3′)	Reverse (5′-3′)
PGRN	TACACCACGGATCTCCTGACCAAG	CAGTGTTGAGGCGGCAGCAG
IL-12	ACCAGAGCAGTGAGGTCTTAGGC	TGTGAAGCAGCAGGAGCGAATG
TNFα	GGTGCCTATGTCTCAGCCTCTT	GCCATAGAACTGATGAGAGGGAG
Arg1	CATTGGCTTGCGAGACGTAGAC	GCTGAAGGTCTCTTCCATCACC
IL-10	CGGGAAGACAATAACTGCACCC	CGGTTAGCAGTATGTTGTCCAGC
PD-L1	AAGCCTCAGCACAGCAACTTCAG	TGTAGTCCGCACCACCGTAGC
β-actin	CATTGCTGACAGGATGCAGAAGG	TGCTGGAAGGTGGACAGTGAGG

### Flow cytometry

The single-cell suspensions were first blocked with anti-CD16/32 (101,302, BioLegend) for 10 min at 4 °C. Then the following antibodies were used: PE/Cy7-PD-L1(124313), APC-CD206 (141707), PE-CD86 (159203), Brilliant Violet 421™-CD86 (105031), APC-CD8a (100711), PE-IFN-γ (505807), PE-Ki-67 (652403), and PE-PD-1 (135205) from Biolegend.

### Orthotopic breast tumor model

Wild-type (WT) C57BL/6 mice aged 6 to 8 weeks were obtained from Chongqing Medical University. PGRN knock out (KO) C57BL/6 mice were a generous gift from Dr. Yibing Yin. Ten WT and ten PGRN KO female C57BL/6 mice were prepared. A total of 5 × 10^6^ PY8119 cells suspended in 100 μl of PBS were injected into the fourth right mammary fat pad of the mice. Subsequently, from the day of modelling, the mental state and activity of mice were observed daily. Tumor growth was evaluated by measuring tumor volume (TV = 0.5 × length×width^2^) every 3 days until they were sacrificed 3 weeks after treatment (*n* = 5, per group). Survival was determined by animal death (*n* = 5, per group). All animal experiments were approved by the Institutional Animal Care and Ethical Committee of Chongqing Medical University.

### Statistical analysis

Unpaired student t-test was used for mean difference comparison between two groups. One-way ANOVA followed by multiple comparison was used for multiple groups. All data were presented as Mean ± SEM (standard error of mean). *P* < 0.05 was considered as significant. All the experiments were performed independently for three times.

## Results

### PGRN promotes the polarization of macrophages towards M2 phenotype

**Fig. 1:**
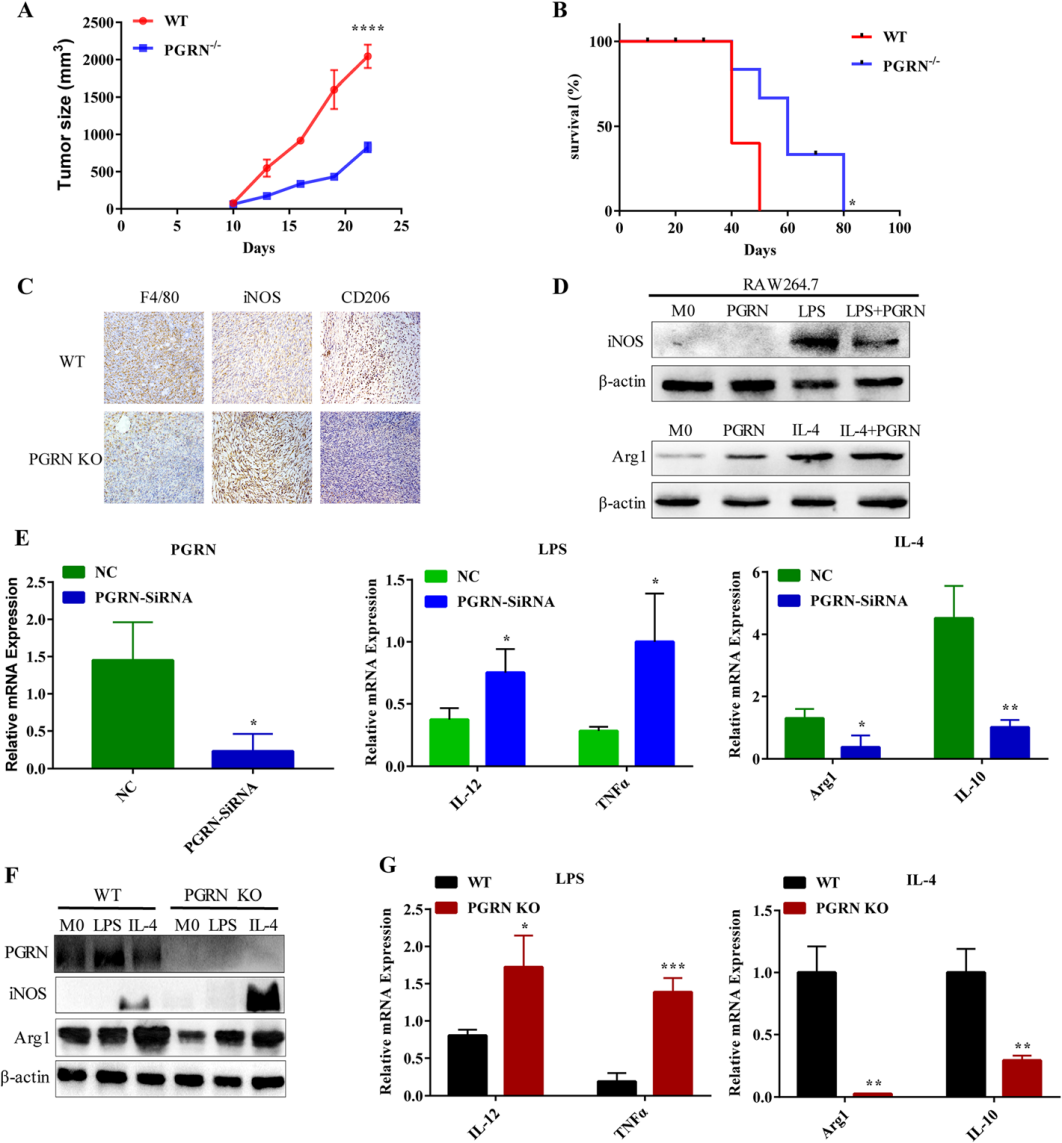
PGRN promotes M2 polarization of macrophages. **a-b**. Breast cancer PY8119 cells were injected in situ into the fat pads of C57 wild-type mice and PGRN knock out mice (*n* = 5 per group). **a**. Tumor volume curve. **b**. Survival curve of mice. **c**. F4/80, iNOS and CD206 expression were detected by IHC in breast cancer tissue sections of WT and PGRN KO mice respectively. **d-e**. RAW264.7 macrophage cell line was treated with PGRN recombinant protein and LPS or IL-4. **d**. iNOS and Arg1 expression were examined by western blot. **e**. M1 markers (IL-12, TNF-α) and M2 markers (Arg1, IL-10) were tested by PCR. (F-G) WT and PGRN KO mouse peritoneal macrophages were treated with LPS or IL-4. **f**. Western blot was performed to analyze iNOS, and Arg1 expression. **g**. The differences in the expression of IL-12, TNF-α and Arg1, and IL-10 were measured by PCR. *p < 0.05; **p < 0.01; ***p < 0.001; ****p < 0.0001

In order to clarify how PGRN affects the growth and metastasis in breast cancer, we first injected breast cancer cells PY8119 into the fat pad of mice in situ to construct a breast cancer model. Compared with the WT group of mice, the breast tumors of PGRN^−/−^ mice grew slower and had better survival (Fig. 1a, b). In contrast, more F4/80 macrophages were infiltrated in the WT group. It is worth noting that the WT group had a low expression of classic M1 marker inducible nitric oxide synthase (iNOS), but a high expression of M2 marker mannose receptor 1 (CD206) (Fig. 1c). Next, we compared the polarization effect of PGRN with the classic inducer LPS and IL-4 on macrophages. We found that PGRN reduced iNOS and CD86 expression, and increased arginase 1 (Arg1) and CD206 expression (Fig. 1d; Supplementary Figure S1A, B). Interestingly, PGRN also reduced the induction of LPS on M1, and the combination of IL-4 and PGRN significantly enhanced the polarization of PGRN on M2 (Fig. 1d). Furthermore, PCR analysis indicated when the PGRN gene expression of RAW264.7 was disturbed, the expression of representative M1 gene *(IL-12)* and *(TNFα)* increased, while the expression of M2 gene *(Arg1)* and interleukin-10 *(IL-10)* decreased (Fig. 1e).

In order to further examine whether endogenous PGRN affects the polarization of macrophages, we treated WT and PGRN^−/−^ peritoneal macrophages with LPS and IL-4 respectively. Interestingly, we found that WT peritoneal macrophages are more sensitive to IL-4 stimulation but not to LPS (Fig. 1f, g). This wasconsistent with the results of the RAW264.7 cell line, indicating that PGRN can promote the M2 macrophages polarization.

### PGRN up-regulates PD-L1 expression on TAMs

**Fig. 2:**
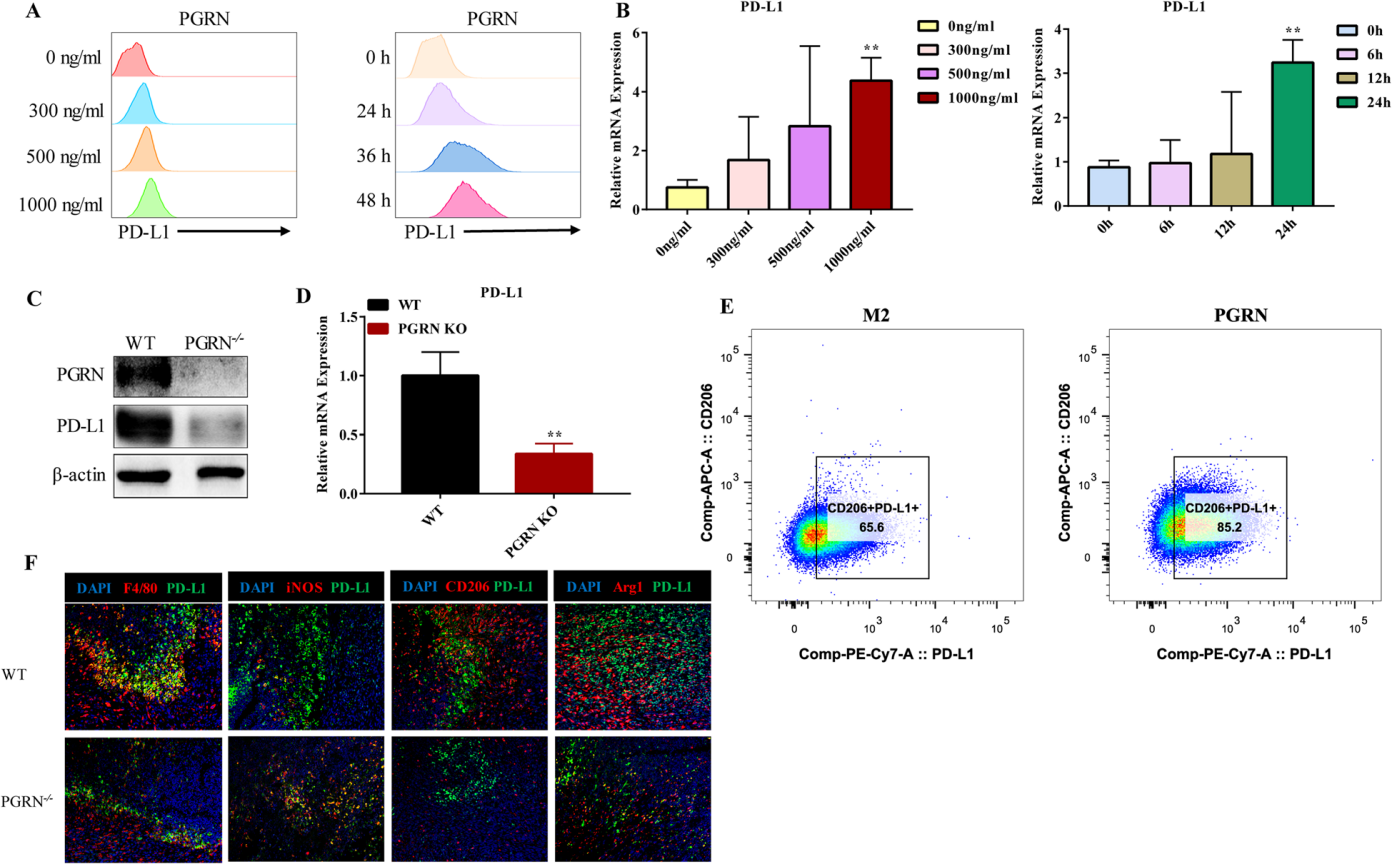
PGRN up-regulates PD-L1 expression on TAMs. **a-b** M2 was treated with PGRN recombinant protein; then PD-L1 expression was measured by flow cytometry and PCR. **c-d**. IL-4 was used to induce WT, PGRN KO peritoneal macrophages into M2; then WB and PCR were used to detect the difference in PD-L1 expression between them. **e**. After being treated with PGRN, the proportion of CD206^+^ PD-L1^+^ cells in M2 were measured by flow cytometry. F. Immunofluorescence was performed to analyze colocalization of F4/80 (red), iNOS (red), CD206 (red), Arg1 (red) and PD-L1 (green) in WT and PGRN KO mice breast cancer sections, and the nucleus was stained with DAPI (blue). **p < 0.01

To ascertain how PGRN affects PD-L1 expression in M2, we first treated M2 with PGRN recombinant protein. Flow cytometry and PCR results showed that PGRN upregulated PD-L1 of M2 in a concentration-dependent and time-dependent manner (Fig. 2a, b). This was also verified by western blot at the same time (Supplementary Figure S2A, B). It is noteworthy that PGRN significantly up-regulated CD206^+^ PD-L1^+^ (Fig. 2e), which further suggested that PGRN did up-regulate PD-L1 of M2. Next, we measured the macrophage markers expression and their respective co-localization with PD-L1 through multicolor immunofluorescence staining. Compared with the PGRN^−/−^ group, F4/80, CD206, and Arg1 expression in the WT group was significantly increased, but the expression of iNOS was lower (Fig. 2f), which was consistent with our IHC results (Fig. 1c). In addition, it was interesting that PD-L1 and F4/80, CD206 and Arg1 in the WT group were significantly co-localized, while the co-localization of iNOS and PD-L1 in the PGRN^−/−^ group were more significant. (Fig. 2f). Then when we treated WT and PGRN^−/−^ peritoneal macrophages with IL-4, we also found that the PD-L1 expression on WT peritoneal macrophages was higher than that on PGRN^−/−^ macrophages, no matter at the protein or the mRNA transcription level. (Fig. 2c, d). The above results indicated that PGRN up-regulated the PD-L1 expression on TAMs.

### PGRN/STAT3 signaling pathway regulates the polarization of TAMs and up-regulates the PD-L1 expression

**Fig. 3:**
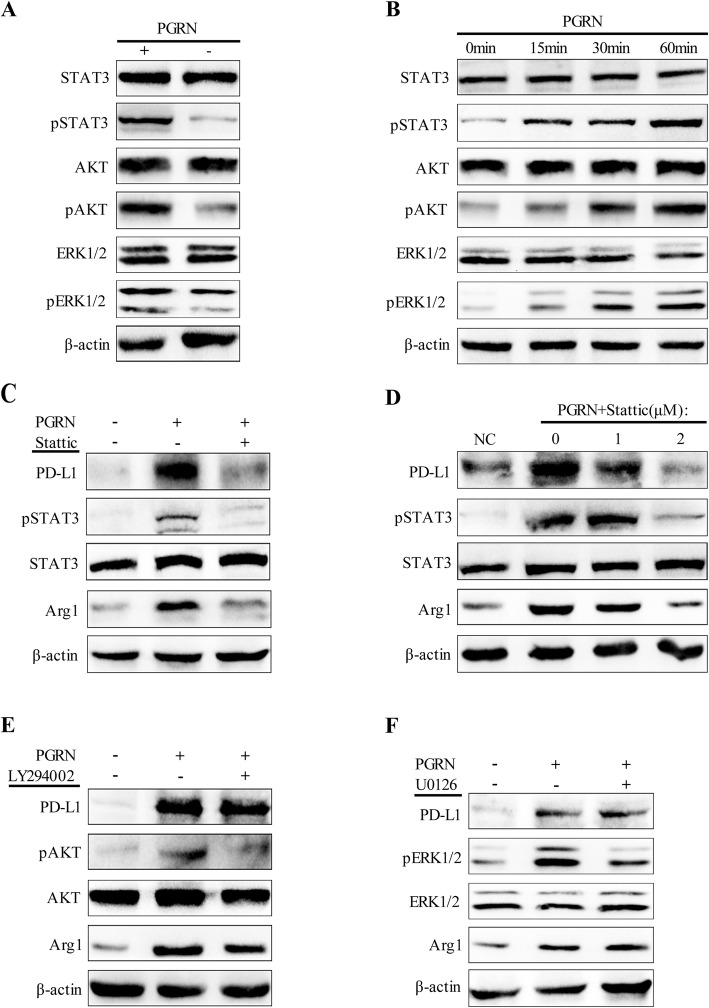
PGRN/STAT3 axis regulates TAMs polarization and up-regulates PD-L1 expression. **a**. After being treated with PGRN, western blot was used to detect STAT3/pSTAT3, AKT/pAKT and ERK1/2/pERK1/2 expression in M2. **b**. M2 was exposed to PGRN at a specified time point, and WB was used to detect the expression of downstream signaling proteins of PGRN. **c-d**. M2 was pretreated with STAT3 inhibitor Stattic, and then PGRN was added. Expression of PD-L1, STAT3/pSTAT3 and Arg1 was examined by Western blotting. **e-f** M2 was pretreated with AKT inhibitor LY294002 and ERK1/2 inhibitor U0126 respectively, and the expression changes of PD-L1, STAT3/pSTAT3 and Arg1 before and after PGRN stimulation were analyzed by Western blot

Next, to explore the signal pathway through which PGRN affects the polarization of TAMs and the PD-L1 expression, we tested the changes of STAT3/pSTAT3, AKT/pAKT and ERK1/2/pERK1/2 signal pathways in TAMs after treatment with PGRN. As expected, WB results showed that PGRN increased phosphorylation of STAT3, AKT and ERK1/2, but had no significant impact on total STAT3, total AKT and total ERK1/2 (Fig. 3a). After exposure to PGRN, phosphorylation of STAT3 was detected at the 15th minute and increased thereafter. In addition, obvious phosphorylation of AKT and ERK1/2 was observed after being stimulated with PGRN for 30 min (Fig. 3b). Next, in order to figure out the key role of STAT3, AKT, and ERK1/2 in PGRN-mediated M2 polarization and PD-L1 expression, we used signaling pathway inhibitors Stattic, LY294002, and U0126 to treat TAMs exposed to PGRN, respectively. Unexpectedly, only Stattic, an inhibitor of STAT3, significantly inhibited the PD-L1 expression in TAMs induced by PGRN, and the inhibition of PD-L1 was enhanced as the dose of Stattic increased (Fig. 3c, d). However, whether LY294002 (AKT inhibitor) or U0126 (ERK1/2 inhibitor) did not distinctly prevent the upregulation of PD-L1 (Fig. 3e, f). And interestingly, after treatment with signal pathway inhibitors, the expression changes of the M2 marker Arg1 were consistent with the PD-L1 response (Fig. 3c-f). These results indicated that PGRN induced TAMs polarization and PD-L1 expression via activating STAT3.

### PGRN promotes CD8^+^ T cell exclusion and inhibits tumor immunity

**Fig. 4:**
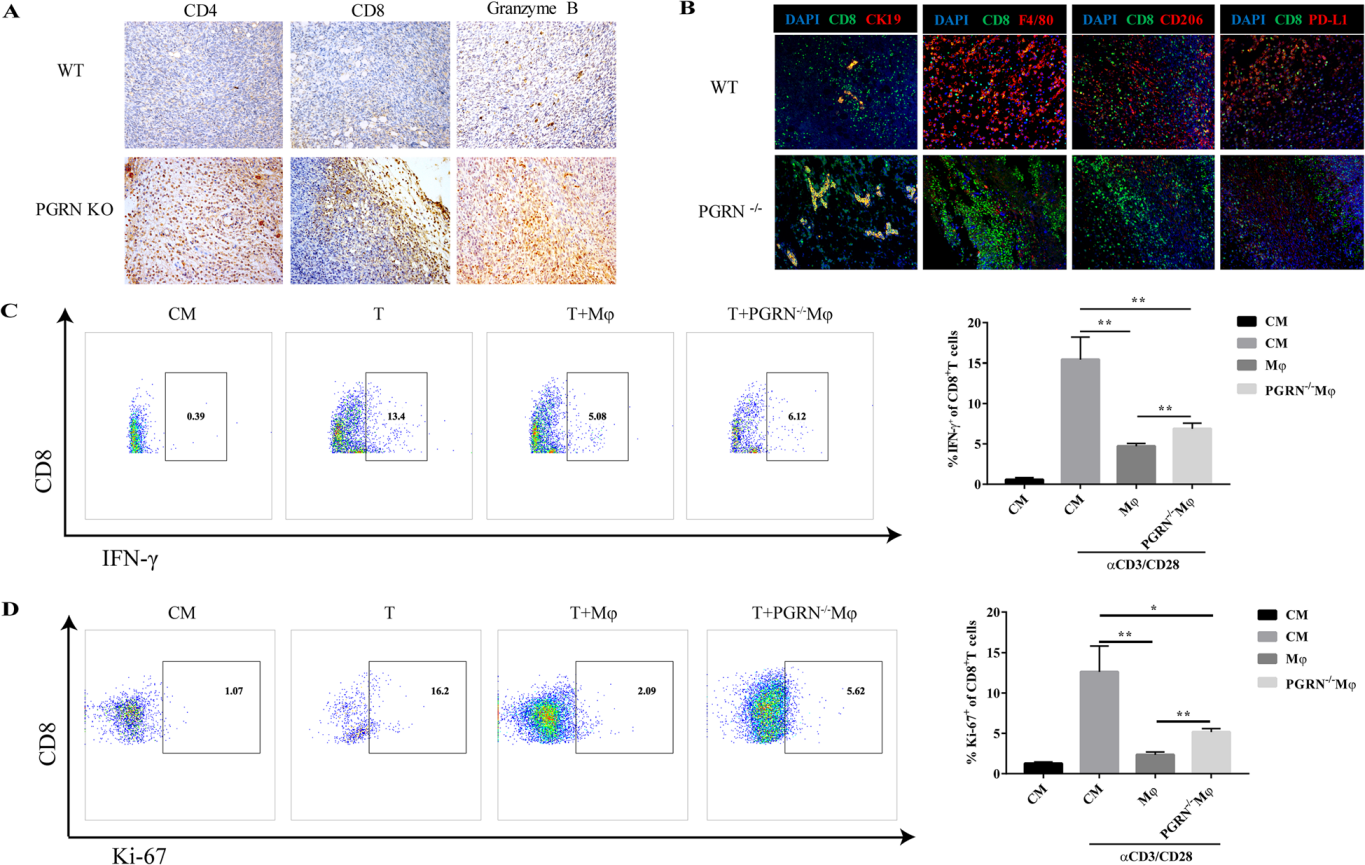
PGRN promotes CD8^+^ T cell exclusion and inhibits tumor immunity. **a**. The expression of CD4, CD8 and Granzyme B in WT and PGRN KO mice breast cancer tissue sections was tested by immunohistochemical staining. **b**. Colocalization of CK19 (red), F4/80 (red), CD206 (red), PD-L1 (red) and CD8 (green) in WT and PGRN KO mice breast cancer tissue sections was examined by immunofluorescence analysis, and the nucleus was stained with DAPI (blue). **c-d** WT and PGRN^−/−^ peritoneal macrophages were co-cultured with mouse spleen lymphocytes activated by αCD3/CD28. Here CM stands for control medium, without αCD3/CD28 stimulation. **c**. Flow cytometry was used to measure the proportion of activated CD8^+^ T cells (IFN-γ^+^ CD8^+^ T cells) and (D) Ki-67^+^ CD8^+^ T cells. *p < 0.05; **p < 0.01

In order to further examine how PGRN affects tumor immune cells, by using immunohistochemical staining, we observed that the infiltration of CD4^+^ cells, CD8^+^ cells, and granzyme B^+^ cells in breast cancer tumor tissues in PGRN^−/−^ group was more than those in WT group, especially CD8^+^ cells and Granzyme B^+^ cells. It is worth noting that the CD8^+^ cells in PGRN^−/−^ group shifted from the tumor stroma to the tumor parenchyma (Fig. 4a). Multicolor immunofluorescence staining analyzed the co-localization of CD8^+^ cells with tumor cells, macrophages, and PD-L1^+^ cells. Interestingly, in WT group, CD8^+^ cells were scattered in the tumor stroma and there was a more obvious co-localization with F4/80^+^ cells, CD206^+^ cells, and PD-L1^+^ cells. Notably, in PGRN ^−/−^ breast cancer tissue sections, the co-localization of CD8^+^ cells and CK19^+^ tumor cells was significantly increased (Fig. 4b). The results above indicated that in breast cancer, PGRN led to CD8^+^ T cell exclusion and affected the spatial distribution of CD8^+^ T cells, which might be related to TAMs or PD-L1 in TME.

Next, to explore whether PGRN affects T cell immune function, we co-cultured the splenic lymphocytes of C57 WT mice with WT or PGRN^−/−^ peritoneal macrophages. It was worth noting that wild type peritoneal macrophages not only inhibited the amount of IFN-γ in CD8^+^ T cells but also inhibited the expression of proliferation antigen Ki-67 by CD8^+^ T cells (Fig. 4c, d). These results indicated that PGRN inhibited tumor immunity in breast cancer via facilitating the exclusion of CD8^+^ T cells.

### PD-1/PD-L1 axis mediates the immunosuppressive function of PGRN in breast cancer

**Fig. 5:**
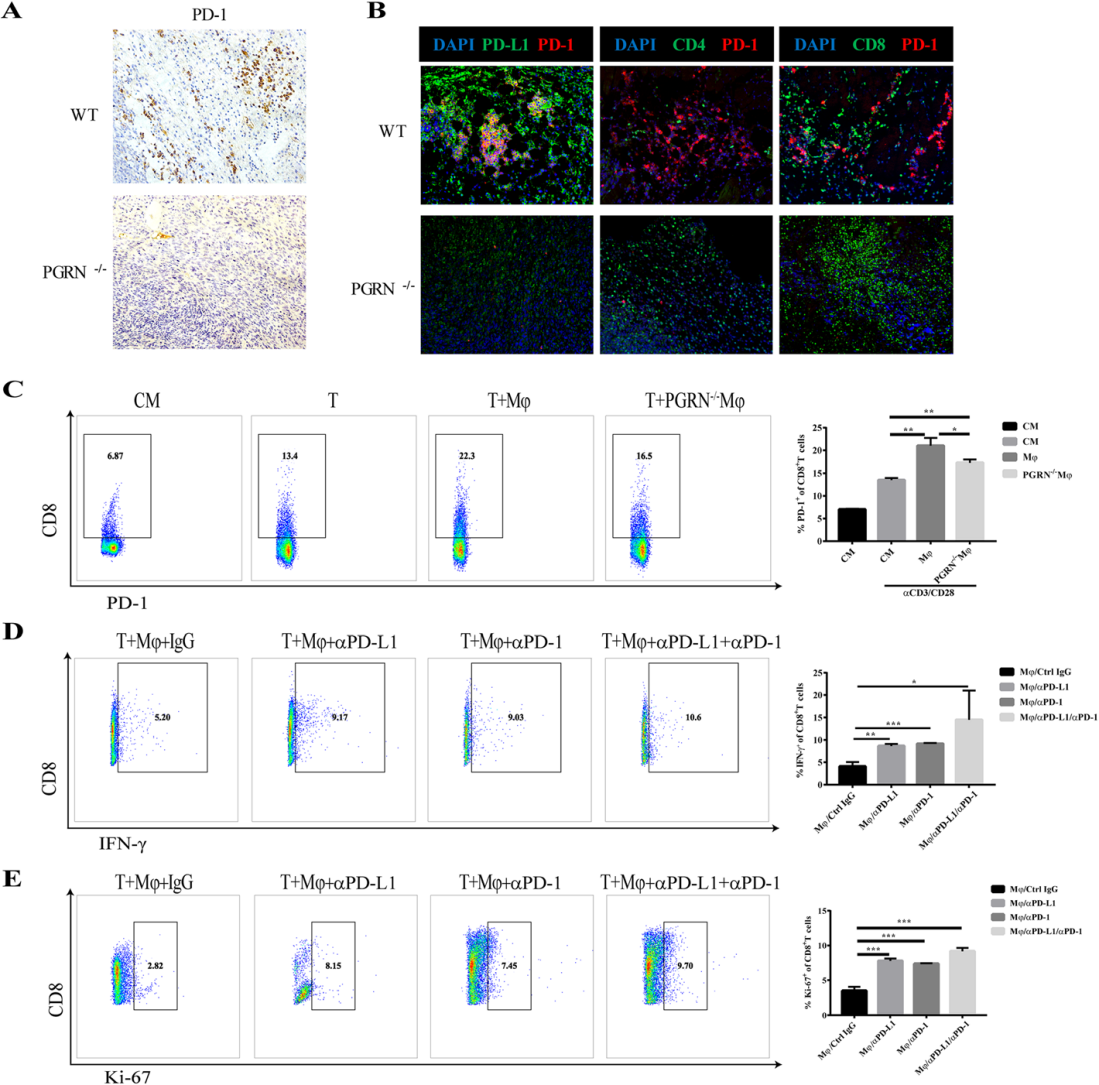
The interaction of PD-1/PD-L1 mediates the immunosuppressive function of PGRN in breast cancer. **a**. Expression of PD-1 in WT and PGRN KO mice breast cancer tissue sections was detected with immunohistochemical staining. **b**. PD-L1 (green), CD4 (green), CD8 (green) expression differences and co-localization with PD-1 (red) in WT and PGRN KO mice breast cancer tissue sections were examined by immunofluorescence, and the nucleus was stained with DAPI (blue). **c**. Mouse splenic lymphocytes activated with or without αCD3/CD28 antibody were co-cultured with WT or PGRN^−/−^ peritoneal macrophages, and the frequency of PD-1^+^ CD8^+^ T cells was tested by flow cytometry. **d-e** Wild-type peritoneal macrophages were co-cultured with splenic lymphocytes preactivated by αCD3/CD28 antibody, and then anti-PD-1 or anti-PD-L1 neutralizing antibodies were added or not to the co-culture system. **d**. CD8^+^ T cell activation and (**e**) CD8^+^ T cell proliferation were detected by flow cytometry. *p < 0.05; **p < 0.01; ***p < 0.001

In the tumor microenvironment, programmed death receptor 1 (PD-1) binds to its ligand PD-L1 to regulate T cell activation and inhibit T cell-mediated immune responses [31, 32]. To explore the molecular mechanism through which PGRN restrained the immune function of CD8^+^ T cells in breast cancer, IHC and immunofluorescence assay were performed. Firstly, we found that PD-1 was highly expressed in WT breast cancer tissues and PD-1 was co-localized with PD-L1 significantly (Fig. 5a, b). Notably, the infiltration of immune cells in WT group was prominently less than that in the PGRN^−/−^ group, especially CD8^+^ cells. And whether it is CD8^+^ cells or CD4^+^ cells, co-localization with PD-1 was identified by immunofluorescence assay (Fig. 5b). When we co-cultured WT and PGRN^−/−^ peritoneal macrophages with splenic lymphocytes of WT mice, the WT group significantly increased the ratio of CD8^+^ PD-1^+^ cells (Fig. 5c). Interestingly, when we added PD-1 neutralizing antibody or PD-L1 neutralizing antibody to the co-culture system, the immunosuppressive function of WT peritoneal macrophages on CD8^+^ cells was distinctly reversed, especially the combination of PD-1 and PD-L1 blockade (Figs. 4 and 5c-d and d, e). The results above indicated that PGRN exerted an immunosuppressive function through PD-1/PD-L1 interaction.

## Discussion

Macrophages contribute more than 50% of the tumor-infiltrating cells [33]. Infiltration of macrophages is closely correlated with poor prognosis of breast cancer [14, 34]. Macrophages regulate their phagocytosis and antigen presentation function through PD-1/PD-L1 immune checkpoints, thereby promoting tumor cells to evade phagocytosis and clearance [35]. In this study, we identified the effect of PGRN on the expression of PD-L1 in macrophages for the first time and demonstrated that PGRN not only promoted the polarization of macrophages to M2 phenotype, but also up-regulated the PD-L1 expression of TAMs.

When we treated TAMs with PGRN, we found that their p-STAT3, p-AKT and p-ERK1/2 protein levels were increased. It has been reported that PGRN interfered with the interaction between TNFα and TNFR by competing with tumor necrosis factor α (TNFα) to bind to tumor necrosis factor receptor (TNFR) [36, 37]. And PGRN activated intracellular protein kinase B (PKB/AKT) and extracellular signal-regulated kinase (ERK) to promote cell proliferation, migration and malignant transformation. Previous studies have shown that PGRN activated ERK and AKT by binding to TNFR1, and activated STAT3 by binding to TNFR2 [38, 39]. Therefore, it may be interesting and meaningful for next research to determine which receptor plays a major role in PGRN-mediated breast cancer immunosuppression. As is known to all, there are various signal pathways concerning the post-transcriptional regulation of PD-L1 [31, 32]. Among these signal pathways, the two most important ones are JAK/STAT/PI3K/AKT/MEK/ERK and SHP2/RAS/RAF/MEK/ERK pathways [40–42]. Therefore, we hypothesized that PGRN regulated the expression of PD-L1 in macrophages through AKT, STAT3 or ERK1/2. To this end, we used inhibitors Stattic, LY294002 and U0126 to block JAK/STAT3, PI3K/AKT and ERK1/2 pathways, respectively. The striking finding was that only after treatment with Stattic, the PD-L1 induction effect of PGRN on TAMs was significantly inhibited, and after the activities of p-AKT and p-ERK1/2 were inhibited, the PD-L1 expression of TAMs did not change significantly. In addition, interestingly, we found that the polarization of M2 was also consistent with the changes in PD-L1. These results indicated that in TAMs, PGRN up-regulated PD-L1 and promoted the polarization of M2 by activating the JAK/STAT3 signaling pathway. Our work in future will further explore whether PGRN regulates this process through other key signal transduction pathways.

Stromal cells in TME, such as cancer-associated fibroblasts (CAF), myeloid-derived suppressor cells (MDSC) and tumor-associated macrophages (TAMs) facilities cancer cells to gain immune privilege through excluding T cells from nearby tumor cells [10, 11, 17]. In our study, we observed that in the breast cancer tissues of the WT group, CD8^+^ T cells were mainly distributed in the tumor stroma, and there was little co-localization with the tumor cell marker CK19. Immunofluorescence results showed that CD8^+^ T cells in the WT group were co-localized with F4/80, CD206, and PD-L1, which implied that TAMs limit the spatial distribution of CD8^+^ T. When splenic lymphocytes were co-cultured with WT and PGRN^−/−^ peritoneal macrophages, WT peritoneal macrophages prominently inhibited the CD8^+^ T cell proliferation and killing activity, indicating that PGRN inhibited breast cancer tumor immunity.

Currently, the PD-1/PD-L1 interaction leading to tumor immune escape is a research hotspot and a difficult point [43], which has not yet been fully explained. Consistent with our observations, it had been reported that in metastatic pancreatic cancer, PGRN induced increased fibroblast infiltration and increased fibrotic matrix formation, resulting in decreased CD8^+^ T cell infiltration and resistance to the αPD-1 antibody therapy. When PGRN was depleted, it could significantly improve the response of metastatic pancreatic tumors to αPD-1 antibody treatment [44]. In this study, when we blocked PD-1 and PD-L1, the immunosuppressive functions of PGRN was distinctly rescued, especially the coadministration of PD-1 and PD-L1 antibody, the ratio of IFN-γ^+^ CD8^+^ T cells and Ki-67^+^ CD8^+^ T cells increased significantly. Taking together, the results of our study show that in breast cancer, PGRN exerts its immunosuppressive function via the PD-1/PD-L1 axis. Next, further researches are needed to explore whether PGRN directly or indirectly affects the PD-1/PD-L1 interaction through other key molecules.

## Conclusions

In this study, through the first investigation of the relationship between PGRN and PD-L1 on macrophages, we have found a new role of PGRN to promote breast cancer progression, that is inducing M2 polarization and up-regulating PD-L1 through PGRN/STAT3, and then promoting CD8^+^ T cell exclusion and inhibiting tumor immunity through PD-1/PD-L1 interaction. This implies that PGRN is expected to become a new therapeutic target for breast cancer immunotherapy, and it may improve the efficacy of clinical immunotherapy in breast cancer via targeting PGRN combined with PD-L1 antibody or PD-1 antibody. In addition, PGRN may become a new clinical diagnostic immunological marker for breast cancer.

## Supplementary Information

**Additional file 1: Figure S1.** PGRN regulates CD86 and CD206 expression on macrophages. **Figure S2.** The expression of PD-L1 on M2 treated with PGRN.

## Data Availability

The datasets analyzed in this study are available from the corresponding author on reasonable request. The datasets supporting the conclusions of this article are included within the article and its additional file.

## Abbreviations

KO: Knock out
PCR: Polymerase chain reaction
PD-L1: Programmed death ligand 1
PD-1: Programmed death receptor 1
PGRN: Progranulin
qPCR: Quantitative PCR
STAT3: Signal transducer and activator of transcription 3
TAMs: Tumor-associated macrophages
TME: Tumor microenvironment
WT: Wild-type
