# Correction to: Progranulin induces immune escape in breast cancer via up-regulating PD-L1 expression on tumor-associated macrophages (TAMs) and promoting CD8^+^ T cell exclusion

**DOI:** 10.1186/s13046-022-02292-7

**Published:** 2022-03-12

**Authors:** Wenli Fang, Ting Zhou, He Shi, Mengli Yao, Dian Zhang, Husun Qian, Qian Zeng, Yange Wang, Fangfang Jin, Chengsen Chai, Tingmei Chen

**Affiliations:** Key Laboratory of Clinical Laboratory Diagnostics (Ministry of Education), College of Laboratory Medicine, Chongqing Medical University, Chongqing, 400016 People’s Republic of China

**Correction to: J Exp Clin Cancer Res 40, 4 (2021)**

**https://doi.org/10.1186/s13046-020-01786-6**

Following publication of the original article [1], the authors identified a minor error in Fig. 3; specifically:

- Fig. 3d Incorrect band used for STAT3; correct image now used

**Fig. 3:**
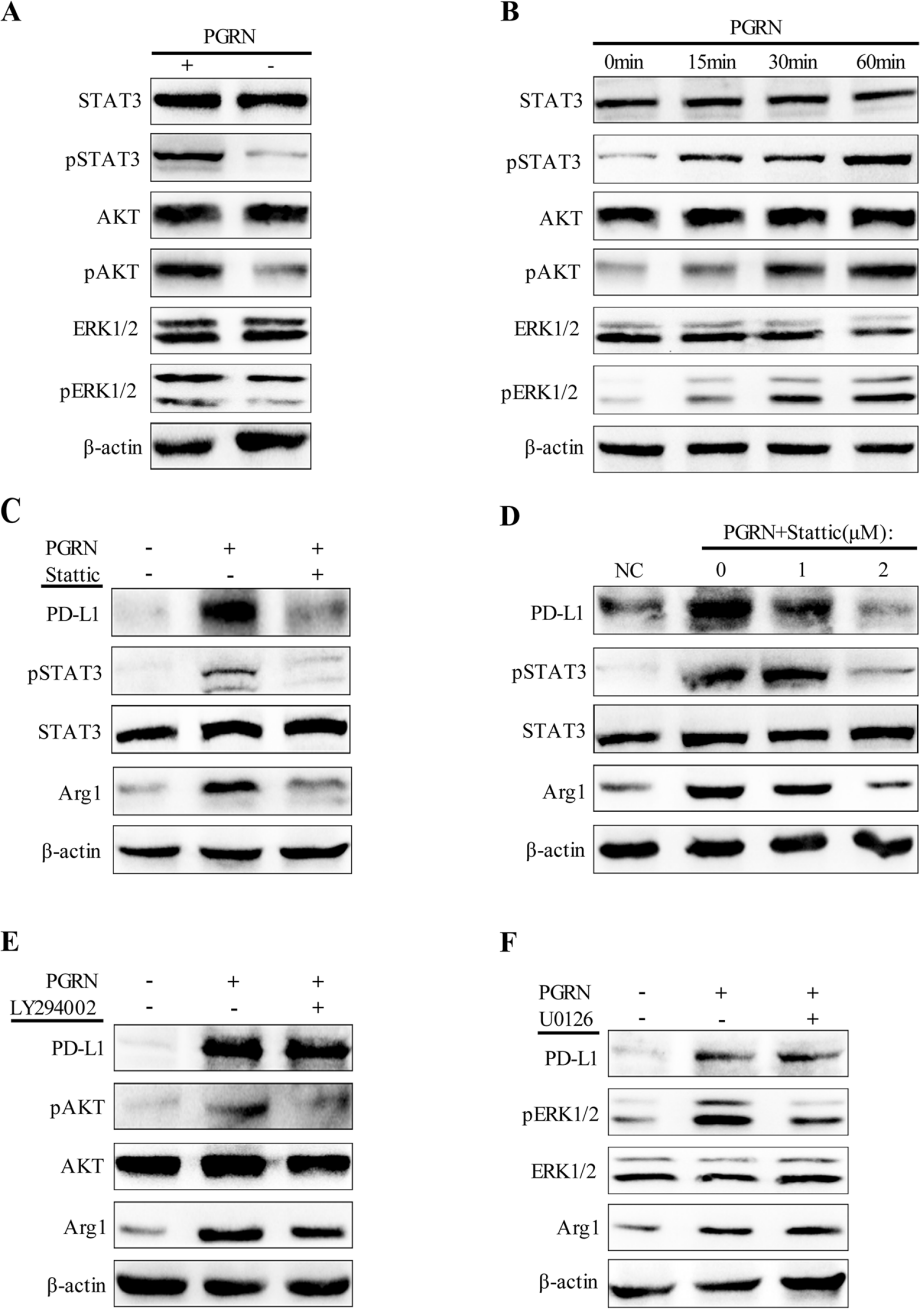
PGRN/STAT3 axis regulates TAMs polarization and up-regulates PD-L1 expression. **a** After being treated with PGRN, western blot was used to detect STAT3/pSTAT3, AKT/pAKT and ERK1/2/pERK1/2 expression in M2. **b** M2 was exposed to PGRN at a specified time point, and WB was used to detect the expression of downstream signaling proteins of PGRN. **c-d** M2 was pretreated with STAT3 inhibitor Stattic, and then PGRN was added. Expression of PD-L1, STAT3/pSTAT3 and Arg1 was examined by Western blotting. **e-f** M2 was pretreated with AKT inhibitor LY294002 and ERK1/2 inhibitor U0126 respectively, and the expression changes of PD-L1, STAT3/pSTAT3 and Arg1 before and after PGRN stimulation were analyzed by Western blot

The corrected figure is given here. In addition, the Supplementary File has been updated to correct the labelling of Fig. S1.

The correction does not have any effect on the final conclusions of the paper. The original article has been corrected.

## Supplementary Information

**Additional file 1: Fig. S1.** PGRN regulates CD86 and CD206 expression on macrophages. **Fig. S2.** The expression of PD-L1 on M2 treated with PGRN.
