# Supplementary material for: Progranulin induces immune escape in breast cancer via up-regulating PD-L1 expression on tumor-associated macrophages (TAMs) and promoting CD8+ T cell exclusion
Source: J Exp Clin Cancer Res. 2021 Jan 4;40:4. doi: 10.1186/s13046-020-01786-6 (PMC7780622; doi:10.1186/s13046-020-01786-6)
Supplement: Supplementary file 1 — Additional file 1: Figure S1. PGRN regulates CD86 and CD206 expression on macrophages. Figure S2. The expression of PD-L1 on M2 treated with PGRN. [file 13046_2020_1786_MOESM1_ESM.docx]

Supplementary Material

Progranulin induces immune escape in breast cancer via up-regulating PD-L1 expression on tumor-associated macrophages (TAMs) and promoting CD8^+^T cell exclusion

Wenli Fang^1^, Ting Zhou^1^, He Shi^1^, Mengli Yao^1^, Dian Zhang^1^, Husun Qian^1^, Qian Zeng^1^, Yange Wang^1^, Fangfang Jin^1^, Chengsen Chai^1^, Tingmei Chen^1^**^*^**

^1^ Key Laboratory of Clinical Laboratory Diagnostics (Ministry of Education), College of Laboratory Medicine, Chongqing Medical University, Chongqing 400016, PR China.

**^*^** Correspondence: tingmeichen@cqmu.edu.cn.

**A**

**
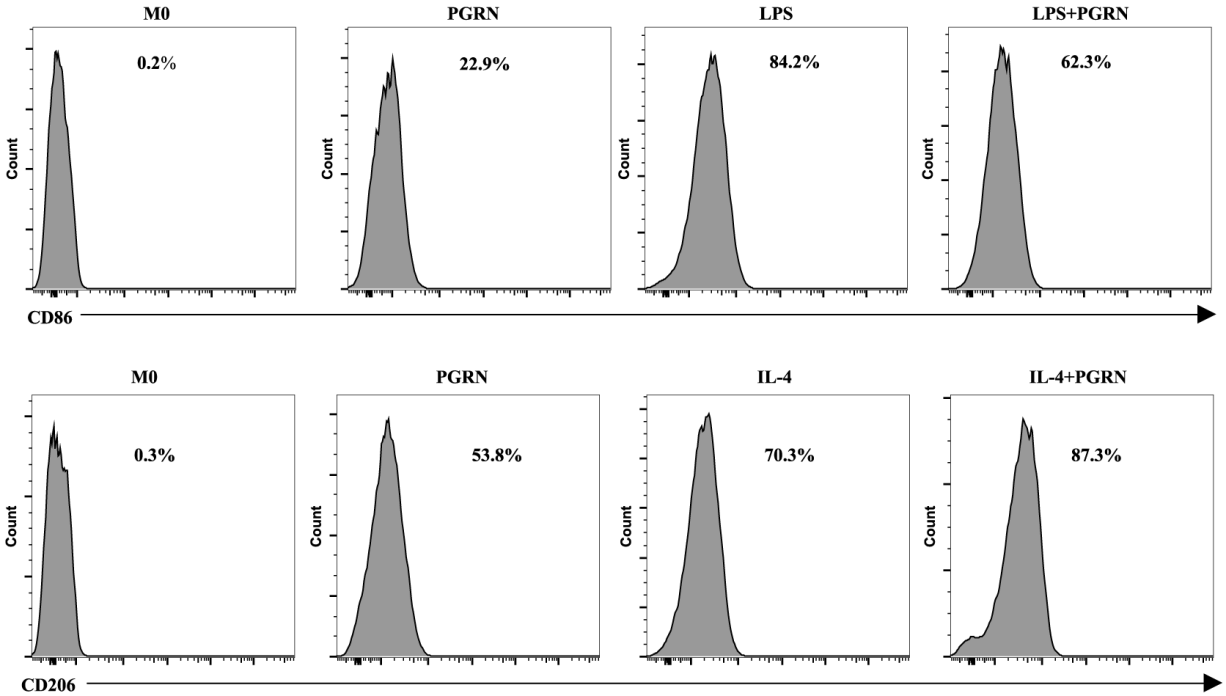
**

**B**

**Fig. S1 PGRN regulates CD86 and CD206 expression on macrophages**

(A-B) RAW264.7 cells were treated with PGRN and LPS or IL-4. CD86 (A) and CD206 (B) expression were tested by flow cytometry. Representative histograms are shown.


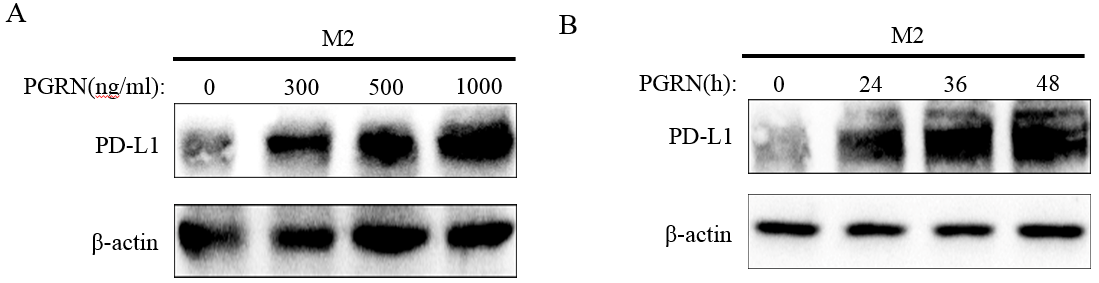


**Fig. S2 The expression of PD-L1 on M2 treated with PGRN**

(A-B) After being treated with PGRN in different dose (A) and time (B), western blotting was used to show PD-L1 expression in M2.
